# Proteomic Analyses Identify Differentially Expressed Proteins and Pathways Between Low-Risk and High-Risk Subtypes of Early-Stage Lung Adenocarcinoma and Their Prognostic Impacts

**DOI:** 10.1074/mcp.RA120.002384

**Published:** 2021-01-26

**Authors:** Juntuo Zhou, Bing Liu, Zhongwu Li, Yang Li, Xi Chen, Yuanyuan Ma, Shi Yan, Xin Yang, Lijun Zhong, Nan Wu

**Affiliations:** 1 Key Laboratory of Carcinogenesis and Translational Research (Ministry of Education), Department of Thoracic Surgery II, Peking University Cancer Hospital & Institute, Beijing, China; 2 Beijing Advanced Innovation Center for Big Data-Based Precision Medicine, Beihang University, Beijing, China; 3 Key Laboratory of Carcinogenesis and Translational Research (Ministry of Education), Department of Pathology, Peking University Cancer Hospital & Institute, Beijing, China; 4 Department of Pathology, Peking University Health Science Center, Beijing, China; 5 Center of Medical and Health Analysis, Peking University Health Science Center, Beijing, China

**Keywords:** lung adenocarcinoma, low-risk subtype, high-risk subtype, proteomics, prognosis, CAF, cancer-associated fibroblast, ECM, extracellular matrix, FASP, filter-aided sample preparation, FDR, false discovery rate, FFPE, formalin-fixed paraffin-embedded, HCD, high-energy collision dissociation, HR, high-risk, IHC, immunohistochemistry, LR, low-risk, LUAD, lung adenocarcinoma, NCE, normalized collision energy, QC, quality control, RFS, recurrence-free survival, TCEP, tris-(2-carboxyethyl) phosphine

## Abstract

The histopathological subtype of lung adenocarcinoma (LUAD) is closely associated with prognosis. Micropapillary or solid predominant LUAD tends to relapse after surgery at an early stage, whereas lepidic pattern shows a favorable outcome. However, the molecular mechanism underlying this phenomenon remains unknown. Here, we recruited 31 lepidic predominant LUADs (LR: low-risk subtype group) and 28 micropapillary or solid predominant LUADs (HR: high-risk subtype group). Tissues of these cases were obtained and label-free quantitative proteomic and bioinformatic analyses were performed. Additionally, prognostic impact of targeted proteins was validated using The Cancer Genome Atlas databases (n = 492) and tissue microarrays composed of early-stage LUADs (n = 228). A total of 192 differentially expressed proteins were identified between tumor tissues of LR and HR and three clusters were identified *via* hierarchical clustering excluding eight proteins. Cluster 1 (65 proteins) showed a sequential decrease in expression from normal tissues to tumor tissues of LR and then to HR and was predominantly enriched in pathways such as tyrosine metabolism and ECM-receptor interaction, and increased matched mRNA expression of 18 proteins from this cluster predicted favorable prognosis. Cluster 2 (70 proteins) demonstrated a sequential increase in expression from normal tissues to tumor tissues of LR and then to HR and was mainly enriched in pathways such as extracellular organization, DNA replication and cell cycle, and high matched mRNA expression of 25 proteins indicated poor prognosis. Cluster 3 (49 proteins) showed high expression only in LR, with high matched mRNA expression of 20 proteins in this cluster indicating favorable prognosis. Furthermore, high expression of ERO1A and FEN1 at protein level predicted poor prognosis in early-stage LUAD, supporting the mRNA results. In conclusion, we discovered key differentially expressed proteins and pathways between low-risk and high-risk subtypes of early-stage LUAD. Some of these proteins could serve as potential biomarkers in prognostic evaluation.

## Highlights

• Proteomic analysis was performed for low-risk and high-risk subtypes of lung adenocarcinoma.
• Key proteins and pathways relevant to low-risk and high-risk subtypes were identified.
• Candidate proteins were evaluated for prognostic impacts in independent cohorts.

## Graphical Abstract

**Figure:**
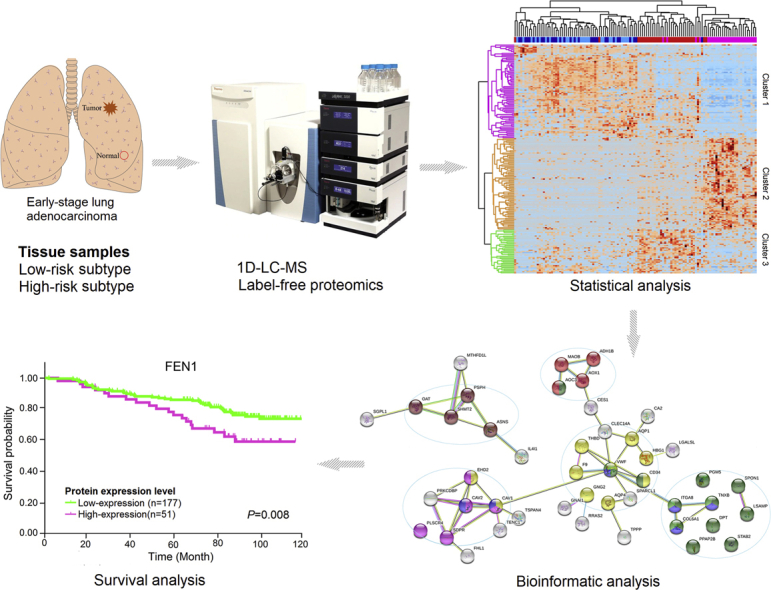

## In Brief

The 1D-LC-MS–based label-free proteomic analysis was performed to analyze the protein expression profiles of early-stage lung adenocarcinoma (LUAD) mainly composed of the low-risk or high-risk subtype. The prognostic impact of certain proteins was validated using other independent cohorts. We discovered key differentially expressed proteins and pathways between low-risk and high-risk subtypes of early-stage LUAD, and some of these proteins could serve as potential biomarkers in prognostic evaluation.

Lung cancer is the leading cause of cancer-related death worldwide, and lung adenocarcinoma (LUAD) is one of the major histological types (1). According to the 2015 World Health Organization Classification of Lung Cancer (2), LUAD is mainly categorized into five histopathological subtypes based on prognosis. Micropapillary and solid patterns are high-risk subtypes with rapid metastatic potential and predict poor prognosis (3, 4). Contrarily, lepidic predominant adenocarcinoma shows slow-growth tendency and has favorable prognosis (5). However, the molecular mechanism underlying this phenomenon is poorly understood. Moreover, invasive adenocarcinomas are usually characterized by coexistence of different histopathological subtypes with varying metastatic capabilities. Further investigation on the evolutionary relationships between these subtypes is required to develop effective treatment and follow-up strategies. Additionally, 30 to 60% of early-stage LUADs will relapse after surgery (6). Therefore, novel efficient biomarkers are required for postoperative monitoring and early detection of recurrence.

Recent advances in proteomic techniques offer powerful tools that can reveal comprehensive protein expression profiles of human tumors. These could be used to elucidate the molecular features and underlying pathogenesis of various tumors and contribute to the development of novel treatment strategies. For instance, proteomic profiling of diffuse-type gastric cancer and pancreatic ductal adenocarcinoma with liver metastases revealed their unique molecular signatures that helped develop an optimal treatment strategy (7, 8). Similarly, proteomic analysis identified novel therapeutic targets of early-stage hepatocellular carcinoma (9).

Several studies have mapped the proteomic landscapes in LUAD. One of them analyzed the proteomic profile of lepidic LUAD and identified some proteins associated with early-stage progression of LUAD (10). Additionally, The Cancer Genome Atlas (TCGA) Research Network published comprehensive molecular profiles of LUAD, including the proteome, focusing on its pathobiology and clinically actionable events (11). More recently, three major proteomic/proteogenomic studies integrated proteome, transcriptome, and genome sequencing data of LUAD and delineated the molecular signature of its pathogenesis and progression. Xu *et al.* (12) revealed three subtypes of LUAD related to clinical and molecular features based on proteomic clustering and potential drug targets were investigated as well. Gillette *et al.* (13) identified multiomic clusters and immune subtypes of LUAD using comprehensive proteogenomic data. Systematical therapeutic candidates were discovered in LUADs featured with driver events. Chen *et al.* (14) focused on the proteogenomic landscape of early-stage and nonsmoking LUAD in East Asia. They also identified three molecular subtypes from multiomic profiles and distinguished clinical features within early stage. These studies provided a comprehensive understanding of LUAD and established molecular classifications in the multiomics level to identify biomarkers or druggable targets. However, histopathological heterogeneity presenting as mixed subtypes within an individual tumor is a prominent feature of LUAD, which makes mechanism interpretation complicated and confounding. Therefore, further studies are required to understand the molecular characteristics of different histopathological subtypes of LUAD and explore the evolutionary relationship between them and related clinical impacts.

In this study, we performed comprehensive proteomic analyses of 59 early-stage LUADs mainly composed of high-risk or low-risk subtype using 1-dimensional LC-MS-based label-free proteomic workflow. We identified some differentially expressed proteins and pathways, including the remodeling of extracellular matrix (ECM) and activation of DNA replication and cell cycle, by comparison of low-risk and high-risk subtypes of early-stage LUAD using bioinformatic analysis. This indicated that these functional proteins and related pathways might be involved in the transition from low-risk to high-risk subtype. Furthermore, some of the differentially expressed proteins were associated with prognosis in LUAD, indicating their clinical relevance as potential biomarkers for postoperative monitoring of early-stage LUAD.

## Experimental Procedures

### Experimental Design and Statistical Rationale

For the 1D-LC-MS-based label-free proteomic analysis, 31 LUAD patients presenting predominantly a low-risk subtype in pathological analysis and 28 cases mainly presenting high-risk subtypes were included. Paired tumor and adjacent normal tissues were collected from each patient, resulting in a total of 118 biological replicates to be analyzed by proteomics. The samples were subjected to LC-MS analysis in a random order. Quality control (QC) samples were prepared by pooling equal volumes of each biological replicate and analyzed every 20 injections during the proteomic analysis.

### Patient Samples

Fifty-nine patients with LUAD who did not receive neoadjuvant therapy and underwent radical resection *via* lobectomy or sublobectomy between January 2013 and December 2017 at the Department of Thoracic Surgery II, Peking University Cancer Hospital & Institute, were enrolled in this study (supplemental Table S1). Two pathologists reviewed the histopathological classification and stage of all patients according to the 2015 World Health Organization Classification of Lung Cancer (2) and the eighth edition of the American Joint Committee for Cancer Staging System for lung cancer (15). The histopathological subtypes, namely lepidic, acinar, papillary, micropapillary and solid patterns, were recorded in 5% increment, and the subtype with the highest percentage was considered as the predominant subtype. Pathological staging T1-2N0M0 was confirmed after surgery. All cases were chosen based on histopathological subtype and categorized into two groups based on the predominant subtype: low-risk subtype group (LR, 31 cases of lepidic predominant adenocarcinomas with more than 50% of lepidic pattern and no micropapillary or solid pattern) and high-risk subtype group (HR, 28 cases of micropapillary and/or solid predominant adenocarcinomas with lepidic pattern as less as possible).The study was conducted in accordance with the Declaration of Helsinki and approved by the Ethics Committee of Peking University Cancer Hospital & Institute (Institutional Review Board No. 2019KT59). All patients provided written informed consent before surgery.

**Fig. 1:**
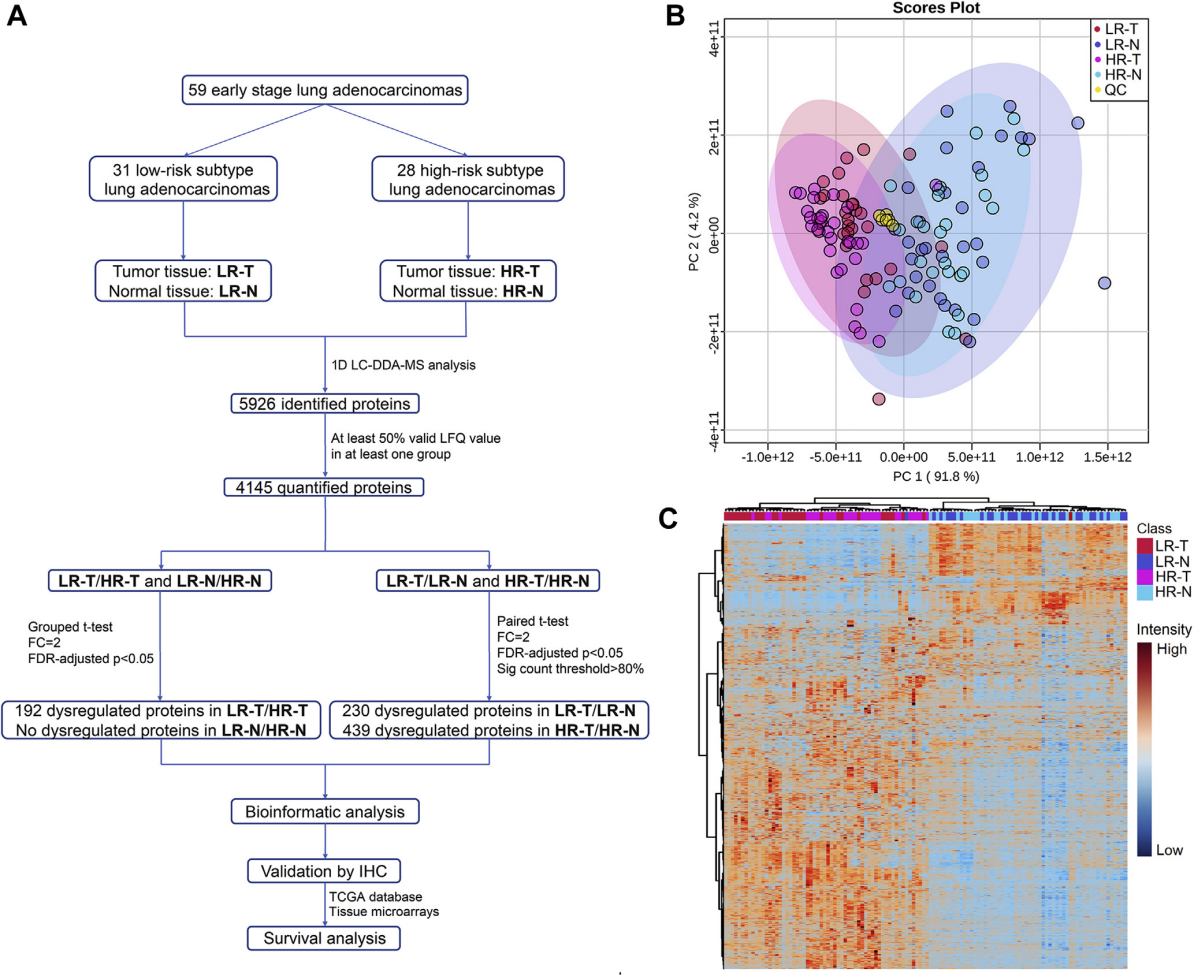
**Overview of the study design and proteomic results.***A*, flowchart of the proteomic analysis. Abbreviations: LR-N, normal tissue of low-risk subtype group; LR-T, tumor tissue of low-risk subtype group; HR-N, normal tissue of high-risk subtype group; HR-T, tumor tissue of high-risk subtype group; QC, quality control. *B*, PCA scatter plot presenting the proteomic result. Groups are presented by colors: *red*, LR-T; *blue*, LR-N; *pink*, HR-T; *light blue*, HR-N; *yellow*, QC. *C*, hierarchical clustering heat map presenting the proteomic result comprised of 5926 identified proteins. Groups are presented by colors: *red*, LR-T; *blue*, LR-N; *pink*, HR-T; *light blue*, HR-N.

Primary tumor tissues and paired normal tissues were collected immediately after resection, then snap-frozen and stored at −80 °C until they were used for the proteomic analysis. Normal tissues were usually collected at the far edge of the resected lobe and macroscopically normal and in two cases of sublobar resection, they were collected more than 2 cm apart from the tumor edge. The experimental samples of each group were further categorized into two subgroups (Fig. 1*A*): LR-T (tumor tissues of low-risk subtype group), LR-N (normal tissues of low-risk subtype group), HR-T (tumor tissues of high-risk subtype group), and HR-N (normal tissues of high-risk subtype group).

### Reagents

Ammonium bicarbonate, sodium deoxycholate, iodoacetamide, and dithiothreitol were purchased from Sigma. Tris-(2-carboxyethyl) phosphine was purchased from Thermo Scientific. Modified sequencing-grade trypsin was purchased from Promega. All of the mobile phases and solutions were prepared using HPLC-grade solvents, including water, acetonitrile, methanol, and formic acid (Sigma Aldrich). Other reagents were from commercial suppliers and of standard biochemical quality.

### Protein Extraction and Trypsin Digestion

Total protein was extracted from tumor and adjacent normal tissue samples. Briefly, frozen tissue samples were homogenized in RIPA lysis buffer (Millipore) containing a protease inhibitor cocktail (Roche). Then, total protein was collected through centrifugation at 12,000*g* for 10 min at 4 °C, and protein concentration was determined using BCA protein assay (Thermo). Protein samples were digested according to the manufacturer’s protocol for filter-aided sample preparation (FASP). Briefly, protein concentrates (500 μg) in an Amicon Ultra-4 10k centrifugal filters (Merck Millipore) were mixed with 500 μl of 8 M urea in 0.1 M Tris/HCl (pH 8.5), and the samples were centrifuged at 13,000*g* at 4 °C for 15 min. The samples were washed twice by adding 500 μl of 8 M urea in 0.1 M Tris/HCl (pH 8.5) and then centrifuged to remove impurities. Further, 20 μl of 0.05 M Tris-(2-carboxyethyl) phosphine (TCEP) prepared in water was added to the filters, and the samples were incubated at 37 °C for 1 h. Then, 20 μl of 0.1 M iodoacetamide (IAA) was added to the filters, and the samples were incubated in the dark for 30 min. The filters were washed twice with 500 μl of 50 mM NH_4_HCO_3_. Finally, 5 μg of trypsin (Promega) in 400 μl of 50 mM NH_4_HCO_3_ was added to each filter with the protein-to-enzyme ratio being 100:1. The samples were incubated overnight at 37 °C, and the released peptides were collected through centrifugation. After drying using vacuum centrifugation, peptides were dissolved in 0.1% formic acid for LC-MS analysis.

### LC-MS Analysis

For proteomics analysis, the samples (1 μg) were analyzed on a home-made C18 column (75 μm × 30 cm, 3 μm) using a U3000 UHPLC connected to a Q-Exactive HF mass spectrometer (Thermo Scientific). The column packing material used was ReproSil-Pur C18-AQ (C18, 3 μm and 120 A°) from Dr Maisch GmbH (Germany). The peptides were separated by a linear gradient from 5 to 35% ACN with 0.1% formic acid at 300 nl/min for 125 min. The MS acquisition was set to DDA mode, including a full MS survey scan from m/z 300 to m/z 1800 at a resolution of 120,000 FWHM (at m/z 200) with AGC set to 5E6 (maximum injection time of 50 ms), followed by 20 MS/MS scans at a resolution of 15,000 FWHM with AGC set to 2E5 (maximum injection time of 100 ms). Twenty of the most intense precursors were selected with an isolation width of m/z 2 for fragmentation *via* high-energy collision dissociation (HCD) with 27 normalized collision energy (NCE). Dynamic exclusion was set to 30 s.

### Protein Identification, Quantification, and Statistical Analysis

Protein identification and label-free quantitation were performed with MaxQuant version 1.5.1.6 using default setting if not stated otherwise. The enzyme was set to trypsin and a maximum of two missed cleavages was allowed. Oxidized methionine (M) and acetylation (protein N-term) were selected as variable modifications, and carbamidomethyl (C) as a fixed modification. Proteins and peptides were identified with a target-decoy approach in revert mode and quantified using intensity data (peak area of extracted ion chromatography) using the Andromeda search engine integrated into the MaxQuant environment. Searches were performed against the UniProt database (Human proteome, downloaded in 2018, 20,399 entries). The mass tolerances on precursor and fragment masses were set at 20 ppm. False discovery rate (FDR) at the protein, peptide, and modification level was set to 0.01. Normalization among samples was performed by the built-in LFQ algorithm in MaxQuant software, and the minimum ratio count for LFQ was set to 2. Match between runs function was enabled and set to 2 min. Proteins with a minimum of two identified peptides were considered as identified, and further more with at least 50% valid values in at least one sample group (LR-T, LR-N, HR-T or HR-N) were considered as quantified. Quantified proteins were subjected to screening for differentially expressed protein without any imputation used to fill in missing values. Statistical analysis for differentially expressed proteins screening was performed using the “Statistical Analysis” tool of Metaboanalyst web service (https://www.metaboanalyst.ca/). Significant *p*-value was determined *via* FDR-adjusted grouped Student’s *t*-test (LR-T *versus* HR-T) or FDR-adjusted paired Student’s *t*-test (LR-T *versus* LR-N and HR-T *versus* HR-N). Differentially expressed proteins were identified using the following criteria: FDR adjusted *p*-value < 0.05, fold change >2, and significant count pair percentage >80% (more than 25 pairs of the 31 pairs reached a fold change of 2 with an accordant trend, exclusively used for paired test). The FDR adjusting procedure for statistical analysis was performed using the built-in algorithm of Metaboanalyst web service.

### Bioinformatic Analysis

PCA score plot and hierarchical clustering (Pearson correlation) based heat map were generated using MetaboAnalyst web service (https://www.metaboanalyst.ca/). Protein–protein interaction (PPI) network construction and Gene Ontology (GO) enrichment analysis were performed using STRING web service (https://string-db.org/) with the differentially expressed proteins revealed by proteomic data used as input. Significantly enriched pathways were retrieved by searching against KEGG and REACTOME databases.

### Immunohistochemistry Analysis

To validate the proteomic results, formalin-fixed paraffin-embedded (FFPE) tissue sections were evaluated for the expression of five proteins (ERO1A, FEN1, CKB, SULT1A3, and CLIC5) *via* immunohistochemistry (IHC) in an independent cohort of 16 early-stage LUADs (eight cases with low-risk subtype and eight cases with high-risk subtype). These five proteins were chosen using the following criteria: (1) Proteins were detected in more than 50% of all samples in the proteomic data; (2) the proteins belonged to cluster 1 or 2, because they presented a sequential change trend from normal tissues to tumors of low-risk subtype group and then to tumors of high-risk subtype group and thus might play important biological functions; (3) the accessibility and performance of antibodies.

As for IHC, sections were dehydrated with graded concentrations of ethanol and immersed in 3% hydrogen peroxide for 15 min. Antigen retrieval was performed by heating for 2 min in a pressure cooker, using 0.01 M citrate buffer (pH 6.0). Sections were then incubated with primary antibodies, namely rabbit polyclonal antibody recognizing human protein ERO1A (1:100, ab177156, Abcam), rabbit polyclonal antibody recognizing human protein FEN1 (1:150, ab109132, Abcam), rat polyclonal antibody recognizing human protein CKB (1:200, ab92452, Abcam), rabbit polyclonal antibody recognizing human protein SULT1A3 (1:150, abx028284, abbexa), or rabbit polyclonal antibody recognizing human protein CLIC5 (1:150, abx033492, abbexa) at 4 °C overnight. The histofine Envision Chem Detection Kit (DaKoCytomation) was used for the secondary antibody at room temperature for 30 min, and 3, 3-diaminobenzidine-hydrogen peroxide was used as the chromogen. Sections were then lightly counterstained with hematoxylin. The primary antibody was substituted with phosphate-buffered saline as a negative control. For evaluation of immunostaining, three representative areas with the most intense immunoreactivity in each tissue section were selected. Staining was graded according to intensity and percentage of positive cells as follows: 0, no staining or <15% positive cells; 1, weak staining in >50% of cells, or moderate staining in 15% to 50% of cells; 2, moderate staining in >50% of cells or strong staining in 15% to 50% of cells; 3, strong staining in >50% of cells.

### Validation of Prognostic Impact in Other Independent Cohorts

In order to evaluate the prognostic impacts of proteins derived from our proteomic analysis, the RNA-seq data set and clinical information for 492 LUADs were firstly obtained from NCI’s Genomic Data Commons (GDC) (https://portal.gdc.cancer.gov/) using R package “TCGAbiolinks” (16), and only “Primary solid Tumor” and “Solid Tissue Normal” samples were included. The downloaded FPKM values were converted to TPM and transformed into log2 (TPM+1). After dividing the patients equally into two groups according to median log-transformed gene expression values, the R package “Survminer” was employed for survival analysis in the Kaplan–Meier estimator, and Cox regression was performed to access the influence of matched mRNA of individual protein on patient survival.

Furthermore, tissue microarrays derived from another independent cohort were used to detect the expression of two representative proteins, ERO1A and FEN1, *via* IHC to evaluate their impacts on patient outcome. This cohort contained 228 early-stage LUADs (T1-2N0M0) who did not receive any antitumor treatment before operation and underwent surgery at the Department of Thoracic Surgery II, Peking University Cancer Hospital & Institute between September 2009 and August 2012. All patients provided written informed consent before surgery. Detailed information on these patients is shown in supplemental Table S2. Staining was graded according to intensity and percentage of positive cells using the scoring criteria described above for IHC analysis, and score = 3 for ERO1A and score ≥ 1 for FEN1 were considered as high expression. Survival analysis was performed using Kaplan–Meier method, and the difference was compared using the log-rank test.

### Statistical Analyses of Clinical Features

Statistical analyses were performed using SPSS 22.0 and GraphPad Prism 7. For analysis of continuous variables, the t-test or Mann–Whitney *U* test was used. The χ^2^ or Fisher’s exact test was used to compare categorical variables. *p* < 0.05 was considered statistically significant.

## Results

### Overview of Proteomic Profiles

To elucidate the difference in the proteomic profiles of LUADs with subtypes of different risk potential, we assembled 59 early-stage LUADs classified into two groups based on their predominant subtype as described in the Materials and methods section: low-risk subtype group (LR, n = 31) and high-risk subtype group (HR, n = 28). The clinical characteristics of the two groups were shown in Table 1. The mean proportion of lepidic pattern in the low-risk subtype group was 90.2%, and the mean proportion of micropapillary and solid patterns in the high-risk subtype group reached a predominant ratio of 59.5%. There were more smokers and males in the high-risk subtype group than in the low-risk subtype group. Patients in the high-risk subtype group were more likely to have stage IB or IIA disease, while the low-risk subtype group was mainly composed of patients with stage IA disease. Moreover, there were more patients with EGFR mutation in the low-risk subtype group, but the difference was not statistically significant. Hierarchical clustering analysis also proved that EGFR mutation is not relevant to the proteomic classification of the subtypes (supplemental Fig. S2). Besides, potential influence of KRAS and ALK mutations could also be excluded as there were only four patients with ALK fusion mutation and one with KRAS mutation in the cohort (supplemental Table S1). Even though there were more patients with stage IB and IIA disease in high-risk subtype group, no significant differences were found among stage IA, IB, and IIA in terms of protein expression profile (supplemental Fig. S3).

**Table 1:** Clinical characteristics of 59 patients

Clinical variable	Low-risk subtype group (n = 31)	High-risk subtype group (n = 28)	*p*^a^
Age (y), mean ± SD	58.48 ± 11.40	59.46 ± 9.10	0.682
Gender, n (%)			0.014
Male	7 (22.6)	15 (53.6)	
Female	24 (77.4)	13 (46.4)	
Smoking, n (%)			0.046
Never smoker	26 (83.9)	17 (60.7)	
Ever/current smoker	5 (16.1)	11 (39.3)	
T stage, n (%)			0.001
1a	5 (16.1)	0 (0)	
1b	14 (45.2)	3 (10.7)	
1c	9 (29.0)	5 (17.9)	
2a	3 (9.7)	16 (57.1)	
2b	0 (0)	4 (14.3)	
TNM stage, n (%)			0.001
IA	28 (90.3)	8 (28.6)	
IB	3 (9.7)	16 (57.1)	
IIA	0 (0)	4 (14.3)	
EGFR mutation			0.091
No	12 (38.7)	17 (60.7)	
Yes	19 (61.3)	11 (39.3)	
Subtype proportion (%), mean ± SD			
Lepidic	90.2 ± 14.0	3.8 ± 7.0	0.001
Acinar + papillary	9.8 ± 14.0	36.3 ± 22.1	0.001
Solid + micropapillary	0 ± 0	59.5 ± 21.1	0.001

a Continuous variables were compared using the Mann–Whitney test and categorical variables were compared using χ^2^ or Fisher’s exact test.

The proteomic profiles of tumor and matched adjacent normal tissues derived from these patients were analyzed, and the study workflow is shown in Figure 1*A*. A total of 5926 proteins were identified with high confidence, and 4145 proteins among them were reliably quantified in no less than 50% samples in at least one group among LR-T, LR-N, HR-T, or HR-N (supplemental Table S3). The score plot of PCA (Fig. 1*B*) and heat map of hierarchical clustering analysis (Fig. 1*C*) were constructed to present an overall view of the proteomic data (5926 proteins). QC samples clustered well in the PCA score plot and samples of tumor and normal tissue separated clearly in both the PCA and heat map.

### Differentially Expressed Proteins Between Low-Risk and High-Risk Subtype Groups

**Fig. 2:**
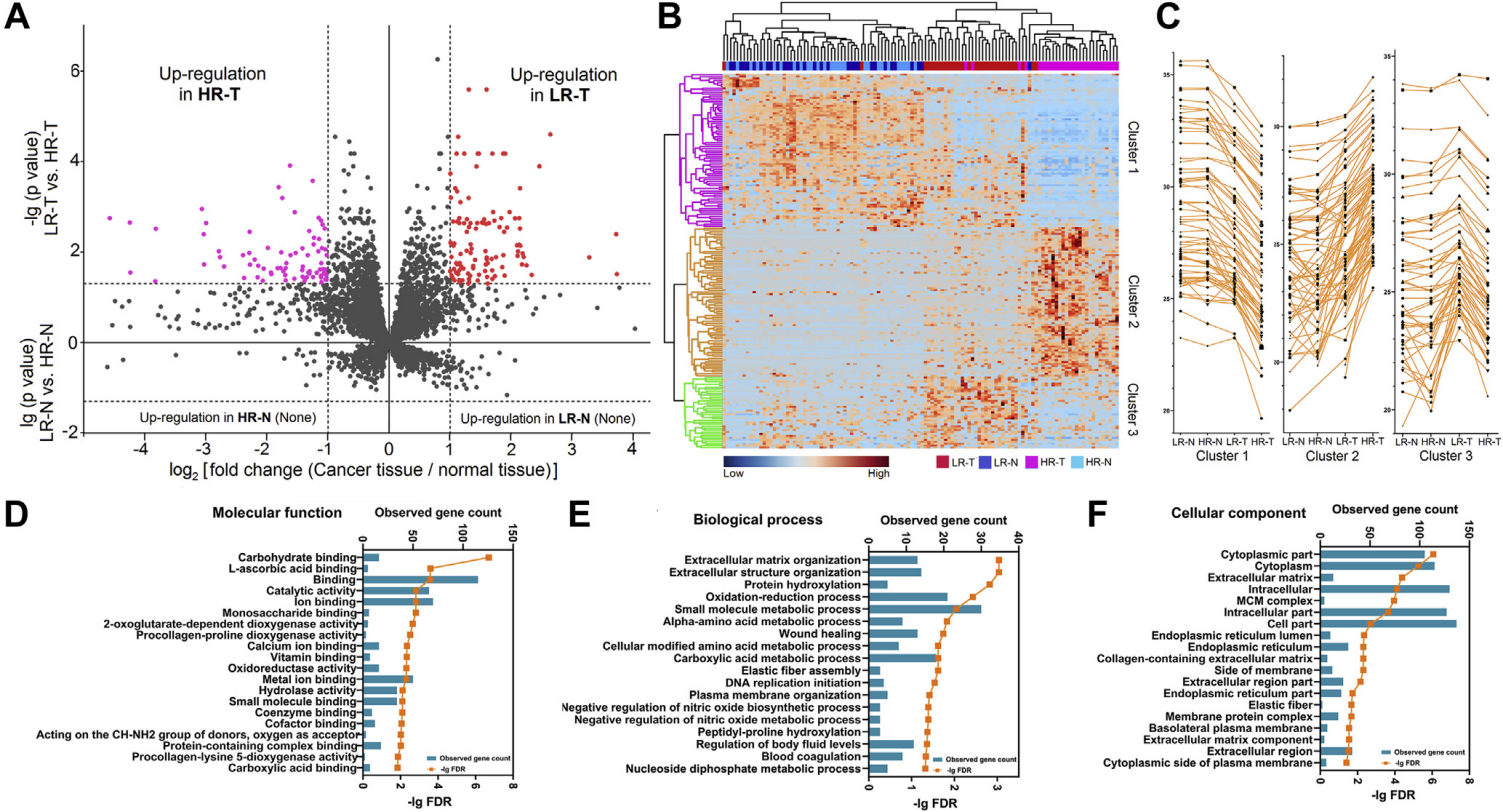
**Differentially expressed proteins between low-risk and high-risk subtype groups.***A*, dual volcano plot presenting the distribution of the differentially expressed proteins between low and high-risk subtype groups. For the *upper part*, the volcano plot presents the comparison result between tumor tissues of low-risk (LR-T) and high-risk (HR-T) subtype groups. The X axis represents the log_2_ (fold change), and the Y axis represents the −lg (*p* value). The upregulated proteins in LR-T are marked in red, and the upregulated proteins in HR-T are marked in purple. For the *lower part*, the volcano plot presents the comparison result between normal tissues of low-risk (LR-N) and high-risk (HR-N) subtypes. The X axis represents the log_2_ (fold change), and the Y axis represents the lg (*p* value). No difference was found between LR-N and HR-N. *B*, hierarchical clustering heat map presenting the expression profile of the differentially expressed proteins in LR-T *versus* HR-T. Three protein clusters were found and presented by colors (*purple*, *orange*, and *green*). Sample groups are labeled by colors: *red*, LR-T; *blue*, LR-N; *pink*, HR-T; *light blue*, HR-N. *C*, line charts presenting the change trends of the three protein clusters. Each polyline represents the level of a differentially expressed protein. *D*–*F*, GO enrichment result of the differentially expressed proteins in LR-T *versus* HR-T in terms of molecular function (*D*), biological process (*E*), and cellular component (*F*). Bars represent the matched gene count (*upper axis*) and orange points represent the -lg (FDR) values (*lower axis*).

We screened for differentially expressed proteins between LR-T and HR-T *via* group *t*-test and identified 192 differentially expressed proteins (supplemental Table S4). No significant difference was observed between the proteomic profiles of LR-N and HR-N. Volcano plots indicating the fold change in expression and the statistical significance for LR-T *versus* HR-T (upper part) and LR-N *versus* LR-N (lower part) are shown in Figure 2*A*. Hierarchical clustering heat map for the 192 differentially expressed proteins was constructed (Fig. 2*B*). Three clusters (labeled as cluster 1–3) containing 184 proteins could be identified and their patterns are indicated using a line chart in Figure 2*C*. Eight proteins were not included in any cluster and also not subjected to bioinformatic analysis because of the insufficient protein number (labeled as NA in supplemental Table S4). Expression of proteins in cluster 1 (65 proteins) decreased sequentially from normal tissues, to LR-T and then to HR-T. In contrast, expression of proteins in cluster 2 (70 proteins) increased sequentially from normal tissues to LR-T and then to HR-T. Additionally, proteins in cluster 3 (49 proteins) showed a higher expression level in LR-T than in normal tissues and HR-T. GO analyses were then performed on the differentially expressed proteins identified in cluster 1 to 3 and the top-ranked GO terms are demonstrated in terms of molecular function (Fig. 2*D*), biological process (Fig. 2*E*), and cellular subtype (Fig. 2*F*). Several GO terms were found to be associated with subtype heterogeneity, such as catalytic activity, ECM/structure organization, substance metabolic processes, and DNA replication. Details of the GO analysis results are listed in supplemental Table S7.

### Differentially Expressed Proteins Between Lung Adenocarcinoma and Normal Tissue

**Fig. 3:**
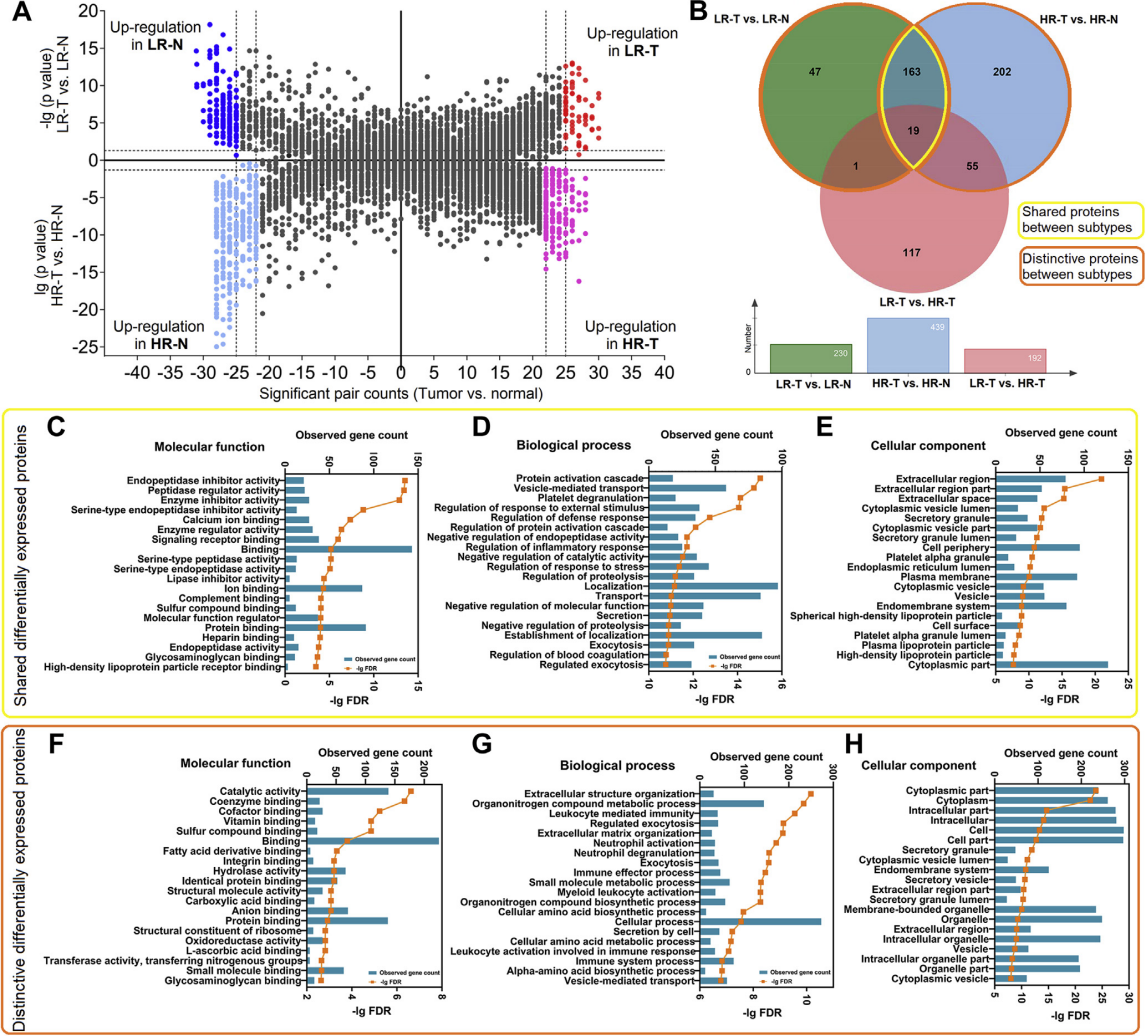
**Differentially expressed proteins between tumor and normal tissues.***A*, dual volcano plot presenting the distribution of the differentially expressed proteins between the tumor and normal tissues of low-risk and high-risk subtype groups. For the *upper part*, the volcano plot presents the comparison result between tumor (LR-T) and normal (LR-N) tissues of low-risk subtype group. The X axis represents the significant pair (a tumor–normal tissue pair that has a fold change >2) counts, and the Y axis represents the −lg (*p* value). The upregulated proteins in LR-T are marked in *red*, and the upregulated proteins in LR-N are marked in *blue*. For the *lower part*, the volcano plot presents the comparison result between tumor (HR-T) and normal (HR-N) tissues of high-risk subtype group. The X axis represents the significant pair counts, and the Y axis represents the lg (*p* value). The upregulated proteins in HR-T are marked in *purple*, and the upregulated proteins in HR-N are marked in *light blue*. *B*, Venn chart presenting the overlap of results revealed by different statistical strategies. LR-T *versus* HR-T was conducted by grouped *t*-test. LR-T *versus* LR-N and HR-T *versus* HR-N were conducted by paired *t*-test. The range of the shared differentially expressed proteins is marked by *yellow*, and the range of the distinctive differentially expressed proteins is marked by *orange*. *C*–*E*, GO enrichment result of the shared differentially expressed proteins between LR-T and LR-N and HR-T and HR-N in terms of molecular function (*C*), biological process (*D*), and cellular component (*E*). *F*–*H*, GO enrichment result of the distinctive differentially expressed proteins in LR-T *versus* LR-N or HR-T *versus* HR-N in terms of molecular function (*F*), biological process (*G*), and cellular component (*H*).

After identifying the differentially expressed proteins between low-risk and high-risk subtype groups, we also investigated the differentially expressed proteins between tumor and matched normal tissues in the two groups as it could provide a more comprehensive proteomic landscape for early-stage LUAD and help understand its molecular features. In low-risk subtype group, we identified 230 differentially expressed proteins between LR-N and LR-T using paired *t*-test (supplemental Table S5). Similarly, 439 proteins were identified between HR-T and HR-N in the high-risk subtype group (supplemental Table S6). Volcano plots in terms of statistical significance and fold change in expression are shown in Figure 3*A*.

Furthermore, 182 proteins were found to be differentially expressed in both tumor tissues of the two groups compared with their matched normal tissues (shared differentially expressed proteins). Additionally, 48 proteins were changed specifically in LR-T compared with LR-N, and 257 proteins were changed specifically in HR-T compared with HR-N as shown in Figure 3*B*. GO analyses were performed using the shared (Fig. 3, *C*–*E*) or distinctive differentially expressed proteins (Fig. 3, *F*–*H*), and the top-ranked terms were represented in terms of molecular function, biological process, and cellular subtype. The shared differentially expressed proteins were mainly associated with inhibitory and regulatory activities, and several proteins were located in the extracellular region. In contrast, the distinctive differentially expressed proteins were primarily related to metabolic, biosynthetic, and immune processes and predominantly located in the cytoplasmic region. Details of the GO analysis results are listed in supplemental Tables S8 and S9.

### Protein–Protein Interaction Network and Perturbed Pathway

**Fig. 4:**
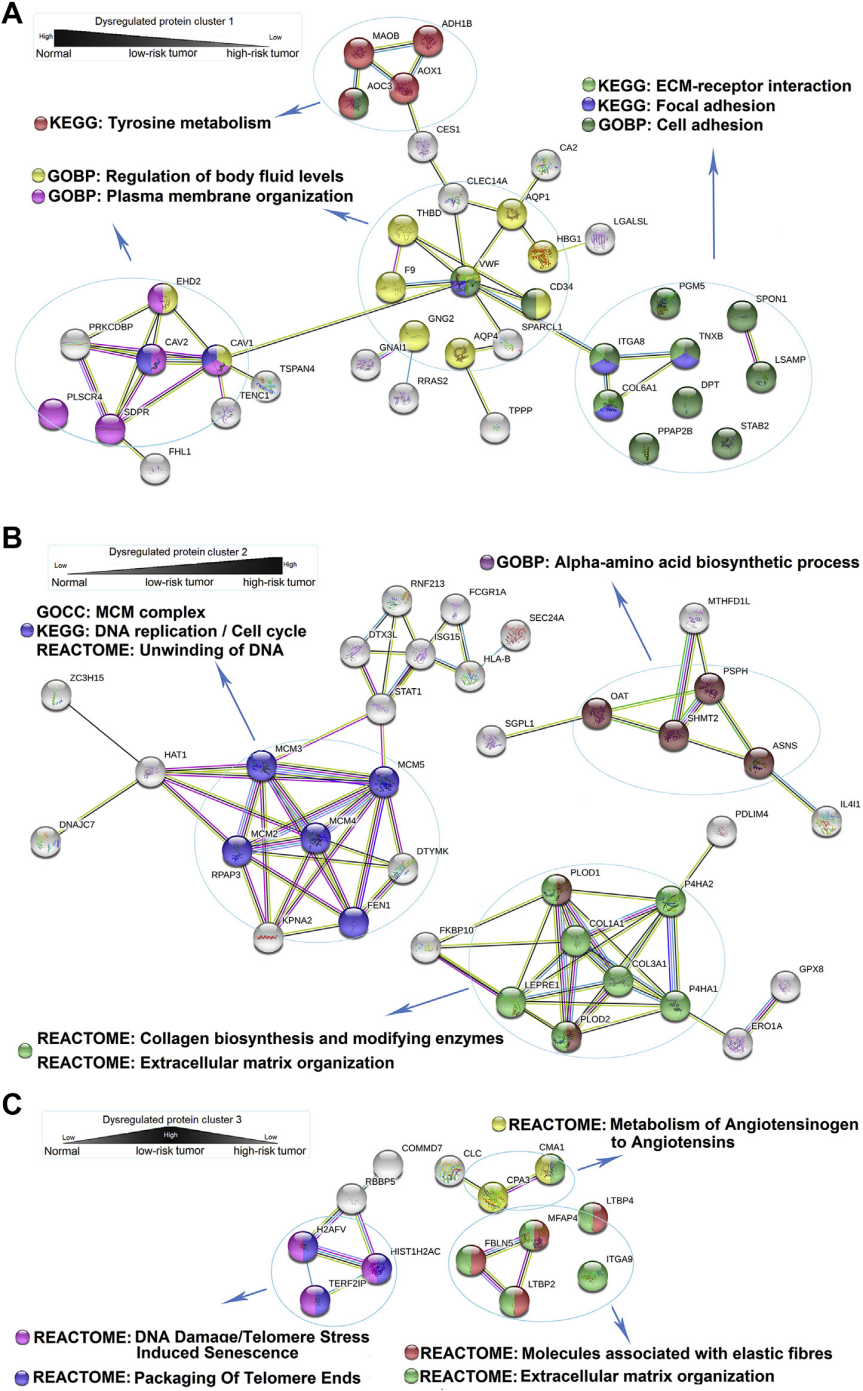
**Protein-protein interaction network of the differentially expressed proteins between tumor tissues of low-risk and high-risk subtype groups.** (*A-C*) Protein-protein interaction network constructed with the differentially expressed proteins in cluster 1 (65 protein inputs; 38 proteins in the network, *A*), cluster 2 (70 protein inputs; 35 proteins in the network; *B*) and cluster 3 (49 protein inputs; 13 proteins in the network; *C*). Enriched pathways or GO terms are marked by colors. Nodes represent proteins and lines represent different interaction categories: light blue, from curated databases; pink, experimentally determined; green, gene neighborhood; red, gene fusions; blue, gene co-occurrence; yellow, text mining; black, co-expression; purple, protein homology.

To construct the PPI network, five groups of differentially expressed proteins were used as input to retrieve corresponding regulatory networks. First, differentially expressed proteins in cluster 1, 2, and 3 (supplemental Table S4) were analyzed separately and the corresponding PPI networks and top-ranked enriched pathways were shown in Figure 4, *A*–*C*. The proteins in cluster 1, the expression of which decreased sequentially from normal tissues, to LR-T and then to HR-T, retrieved a network comprising 38 proteins. These proteins were mainly enriched in pathways such as tyrosine metabolism (*e.g.*, MAOB, AOC3, AOX1, ADH1B), ECM–receptor interaction, and focal adhesion (*e.g.*, ITGA8, TNXB, COL6A1). In contrast, the proteins in cluster 2, the expression of which increased sequentially from normal tissues to LR-T and then to HR-T, retrieved a network comprising 35 proteins. These proteins were mainly enriched in pathways such as ECM organization (*e.g.*, COL1A1, COL3A1, P4HA2, P4HA1, PLOD1, LEPRE1, PLOD2), DNA replication, and cell cycle (*e.g.*, MCM2, MCM3, MCM4, MCM5, DTYMK, FEN1, KPNA2). The proteins in cluster 3, the expression of which only increased in LR-T, retrieved a network comprising 13 proteins. These proteins were significantly enriched in pathways such as metabolism of angiotensinogen to angiotensin (*e.g.*, CMA1 and CPA3), telomere stress-induced senescence, and packaging of telomere ends (*e.g.*, H2AFV, HIST1H2AC, and TERF2IP).

**Fig. 5:**
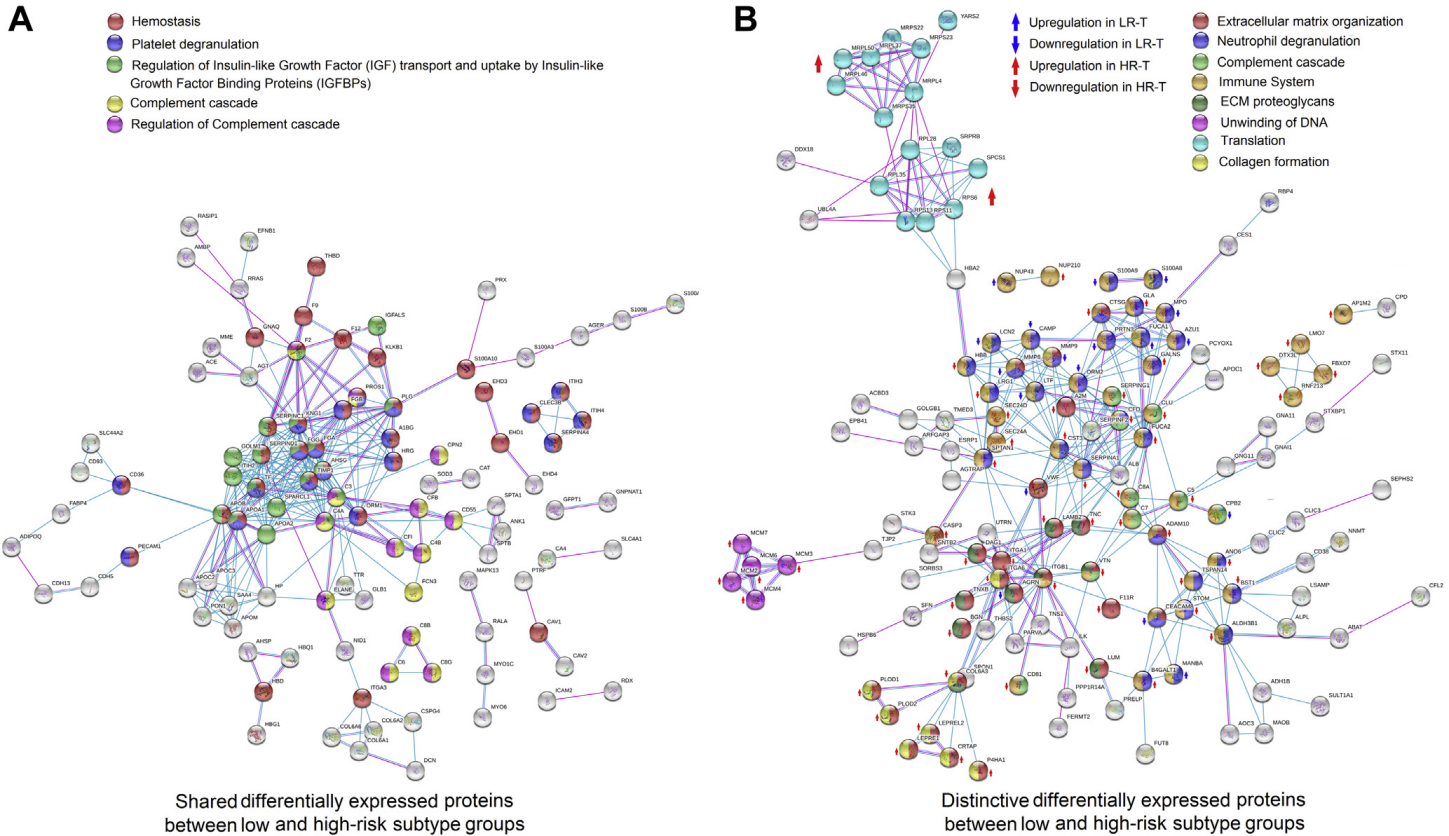
**Protein–protein interaction network of the shared or distinctive differentially expressed proteins between low-risk and high-risk subtype groups.***A* and *B*, protein–protein interaction network constructed with the shared differentially expressed proteins (182 protein inputs; 104 proteins in the network, *A*) or distinctive differentially expressed proteins (305 protein inputs; 140 proteins in the network, *B*) between low-risk and high-risk subtype groups. Enriched pathways or GO terms are marked by colors. Nodes represent proteins and lines represent different interaction categories: *light blue*, from curated databases; *pink*, experimentally determined; *green*, gene neighborhood; *red*, gene fusions; *blue*, gene co-occurrence; *yellow*, textmining; *black*, coexpression; *purple*, protein homology. Change trend and subtype specificity of the distinctive differentially expressed proteins are marked by *colored arrows*.

The shared and distinctive differentially expressed proteins shown in Figure 3*B* (shared and distinctive proteins between supplemental Tables S5 and S6) were separately sorted out as the fourth and fifth groups of proteins for PPI network construction (Fig. 5, *A* and *B*). The shared differentially expressed proteins (182) retrieved a network comprising 104 proteins, which were enriched in pathways such as hemostasis, platelet degranulation, and complement cascade. Similar to the results presented in Figure 4, which demonstrated different features between low-risk and high-risk subtype group, the distinctive differentially expressed proteins (305) retrieved a network comprising 140 proteins, which were also mainly related to ECM organization, immune system, translation, etc. Details of the enriched pathways are listed in supplemental Tables S10–S12.

### Validation of the Proteomic Results by Immunohistochemistry

**Fig. 6:**
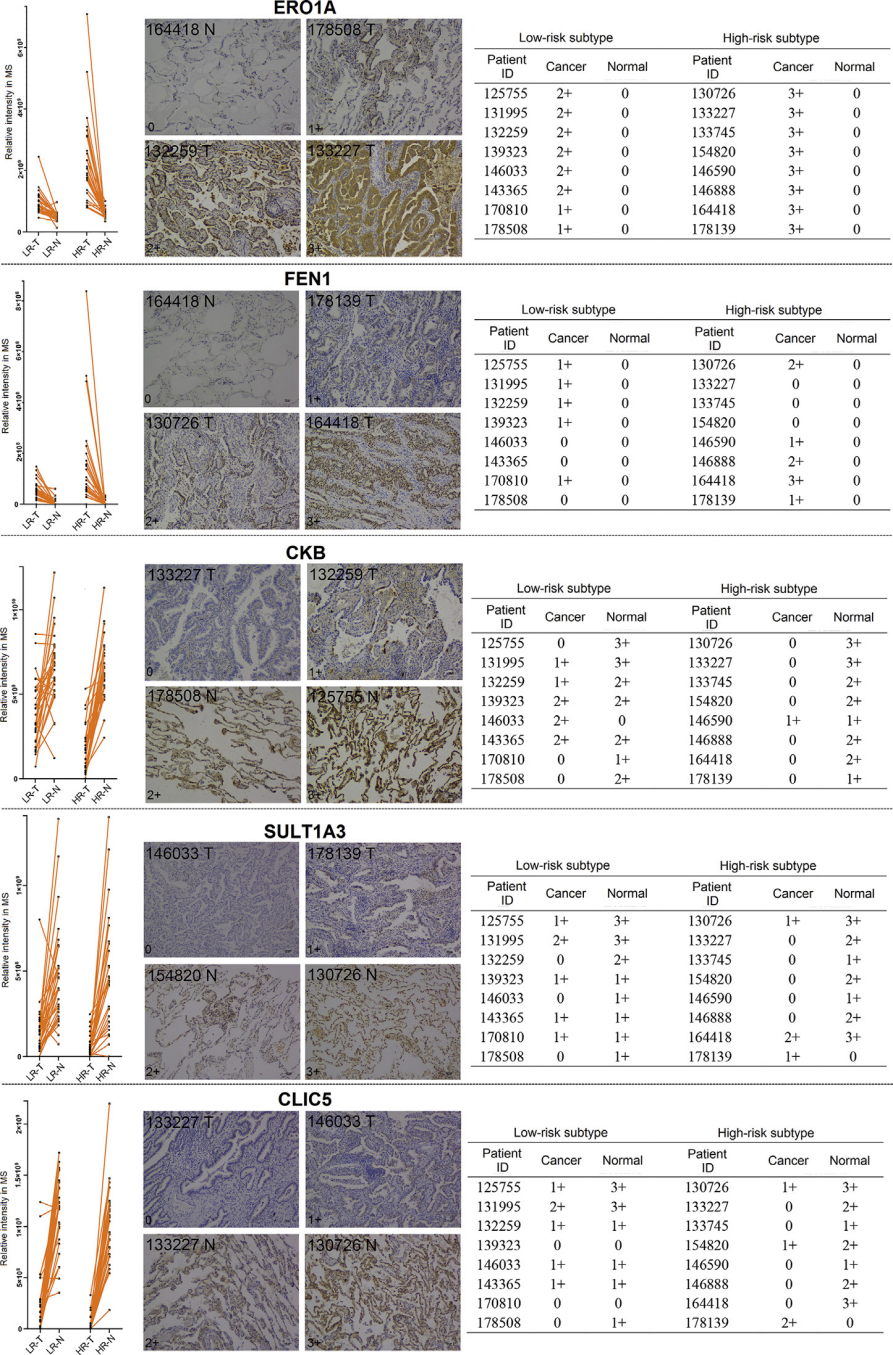
**Immunohistochemistry analysis of the five selected differentially expressed proteins.** Line chart of the proteomic result, immunohistochemistry grading examples and immunohistochemistry grading result table are presented from left to right for each target protein. Staining was graded according to intensity and percentage of positive cells as follows: 0, no staining or <15% positive cells; 1, weak staining in > 50% of cells, or moderate staining in 15% to 50% of cells; 2, moderate staining in > 50% of cells or strong staining in 15% to 50% of cells; 3, strong staining in >50% of cells.

To validate the proteomic results, five differentially expressed proteins were selected from cluster 1 (CKB, SULT1A3, and CLIC5) and cluster 2 (ERO1A and FEN1). Their expression levels were detected *via* IHC in an independent cohort containing eight low-risk and eight high-risk subtype LUADs. The line chart of proteomic results, the representative IHC images, and overall result of IHC grading scores were arranged from left to right for each selected protein (Fig. 6). The expression trends of these five proteins could be clearly observed according to the grading results, and they were in accordance with the proteomic result.

### Expression of Some Differentially Expressed Proteins May Be Correlated With Prognosis of Lung Adenocarcinoma

**Fig. 7:**
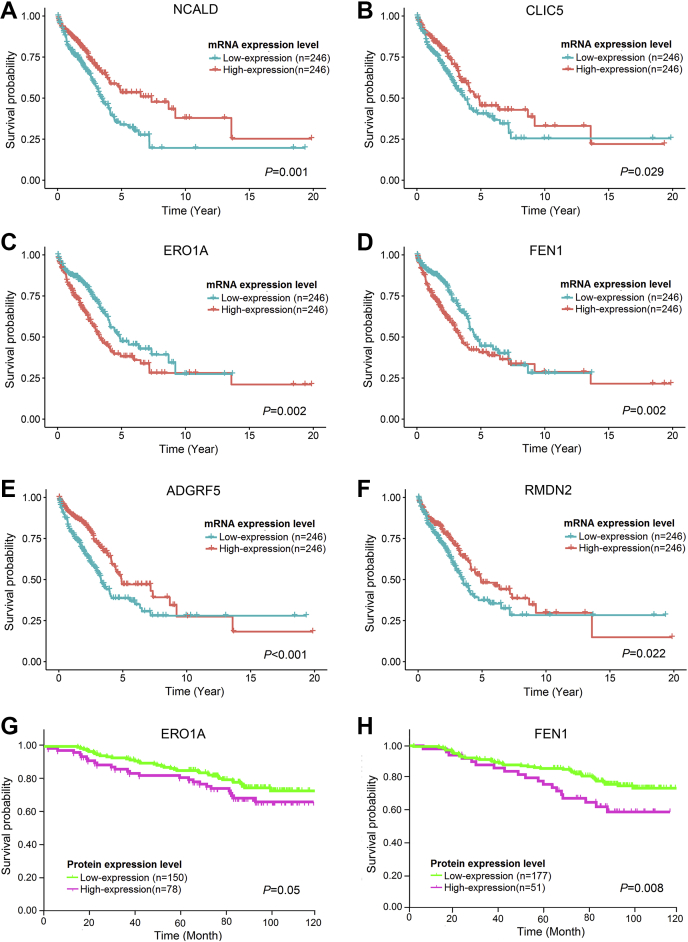
**Overall survival analysis** (*A-F*) Overall survival analysis of the matched mRNAs of the differentially expressed proteins in cluster 1-3 using RNA-seq dataset and survival information of 492 lung adenocarcinoma patients from TCGA. The Kaplan-Meier curves of representative proteins in cluster 1 (*A-B*), cluster 2 (*C-D*) and cluster 3 (*E-F*) are presented. (*G-H*) Overall survival analysis by the protein expression levels of ERO1A (G) and FEN1 (H) using immunohistochemical staining in tissue microarrays containing 228 early-stage lung adenocarcinoma patients. The Kaplan-Meier curves are presented. Staining was graded according to intensity and percentage of positive cells as follows: 0, no staining or <15% positive cells; 1, weak staining in > 50% of cells, or moderate staining in 15% to 50% of cells; 2, moderate staining in > 50% of cells or strong staining in 15% to 50% of cells; 3, strong staining in >50% of cells. Score=3 for ERO1A and score≥1 for FEN1 were considered as high expression.

It has been demonstrated that LUADs with high-risk subtype showed poorer prognosis than those with low-risk subtype (5, 17). We hypothesized that some of the 184 differentially expressed proteins identified in clusters 1 to 3 might be associated with the prognosis of LUAD. We obtained RNA-seq data set and survival information for 492 LUADs from TCGA database and then screened the matched mRNAs of the 184 proteins. Finally, 63 mRNAs were found to be significantly correlated with the overall survival (*p* < 0.05, supplemental Table S13), including 18, 25, and 20 mRNAs in clusters 1, 2, and 3, respectively. Notably, high expression of the 25 matched mRNAs of proteins in cluster 2 predicted poor prognosis. In contrast, high expression of the 18 and 20 matched mRNAs of proteins in cluster 1 and 3 was associated with a favorable outcome. The Kaplan–Meier curves of 6 representative mRNAs from cluster 1 (NCALD and CLIC5), cluster 2 (ERO1A and FEN1), and cluster 3 (ADGRF5 and RMDN2) are shown in Figure 7, *A*–*F*. Additionally, we performed IHC to detect the expression of ERO1A and FEN1 (the high expression of matched mRNAs of these proteins was associated with poor prognosis) in tissue microarrays, which contained 228 cases of LUADs with pathological stage T1-2N0M0. According to the IHC scoring, high expression of ERO1A (IHC score = 3, *p* = 0.05) and FEN1 (IHC score ≥ 1, *p* = 0.008) was correlated with shorter overall survival, which showed prediction power similar to their matched mRNA level in prognostic evaluation (Fig. 7, *G* and *H* and supplemental Table S14).

## Discussion

Histological heterogeneity is a typical feature of LUAD and is associated with its prognosis. Micropapillary and solid patterns are high-risk subtypes and are correlated with poor prognosis. Whereas lepidic pattern is considered as low-risk subtype predicting favorable prognosis. Molecular mechanism leading to these diversities is still ambiguous. Previous studies indicated that EGFR mutations were more common in lepidic pattern than other subtypes, which was also verified in our study. Likewise, recent studies revealed that solid predominant LUAD displayed an active cytotoxic immune signature, which suggested that such patients could benefit from immunotherapy (18, 19, 20). These studies mainly revealed features of different subtypes at genome and transcriptome levels. As proteins are considered as the “determinants of phenotype,” the characterization of protein profiles can help to better understand the molecular features of different subtypes of LUAD. In this study, we performed comprehensive proteomic analyses of 59 LUADs with low-risk or high-risk subtype for the first time. In total, more than 4000 reliably quantified proteins were retrieved and the proteomic results were reliable and of great quality as indicated in the score plot of PCA and heat map of hierarchical clustering analysis (Fig. 1).

We first revealed differentially expressed proteins between tumor tissues of low-risk and high-risk subtype groups, which were further categorized into three protein clusters (Fig. 2). Proteins in cluster 1 and 2 showed continuous change trends from normal tissues to LR-T and then to HR-T. It reminded us that there might be a transitional relationship between low-risk and high-risk subtypes and these proteins might play important roles in this process. A previous study also reported lineage relationship between lepidic and nonlepidic patterns in LUAD by detecting genomic rearrangements (21). Proteins in cluster 3 specifically showed high expression in LR-T. It may represent unique features of LUAD with low-risk subtype and can serve as potential biomarkers for subtype diagnosis. Furthermore, a separate comparison between tumor and matched normal tissues of these two subtype groups revealed shared and distinctive proteins that changed in these two subtypes (Fig. 3). The shared proteins may represent the common features and pathogenesis of different LUAD subtypes, which were, as the GO results showed, significantly relevant to enzyme inhibitor activity and located at extracellular region. While the distinctive proteins represent the different features between subtypes, which were significantly relevant to molecular-binding functions and immune responses. These clues provide a relatively comprehensive understanding of the mechanism involved in LUAD tumorigenesis and subtype differentiation. Similar to previous reports (22, 23), there were more smokers in the high-risk subtype group. Recent studies indicated that the pathogenesis of LUADs in smokers and nonsmokers might be different. However, there was no difference of protein expression between normal tissues of the two groups. This indicated that smoking had insignificant impact on the expression of proteins in LUAD and that it may be mainly related to gene mutation of LUAD (24, 25).

Three recent high-impact proteomic/proteogenomic studies integrated multiomics data of LUAD to explore its biology and potential therapeutic targets. Gillette *et al.* (13) revealed four subtypes of LUAD defined by key driver mutations, country, and gender. In their study, proteomic and phosphoproteomic data illuminated downstream biology of copy number aberrations, somatic mutations, and fusions and identified therapeutic vulnerabilities associated with driver events. Different from their study, Chen *et al.* (14) focused on early-stage, predominantly female, and nonsmoking lung adenocarcinoma and illuminated the molecular phenotype of this demographically distinct disease. Xu *et al.* (12) stratified LUAD into three proteomic subtypes, and they were designated S-I (environment and metabolism high), S-II (mixed type), and S-III (proliferation and proteasome). Among them, S-I was linked with favorable outcome and enriched with early-stage LUADs and lepidic pattern, whereas S-III tended to be more prognostic and correlated to lower degree of differentiation and more solid pattern.

Different from three aforementioned studies in methodology, we recruited two groups of LUAD patients with different histopathological subtypes, one group predominantly presenting lepidic pattern *versus* another predominantly with solid/micropapillary pattern and compared their proteomic profiles. Based on this strategy, we disclosed key proteins or pathways during tumor progression from low-risk pattern to high-risk pattern and identified three clusters reflecting a sequential changing tendency from normal tissue to low-risk subtype and then to high-risk subtype, which might facilitate biomarker selection for prognostic evaluation or early detection of recurrence. Our study provided direct evidence of prognosis-related proteomic subtype classification based on distinct histopathological phenotypes. Compared with Xu’s study, nine proteins in cluster 1 of this study indicating favorable outcome were also highly expressed in Xu’s S-I cluster, such as MOAB, TMOD2, and ABCA8. Similarly, ten proteins in cluster 2 with poor prognosis were highly expressed in Xu’s S-III cluster, including PLOD2, SLC16A3, and TUBB3. We speculated that these common proteins identified in different studies suggested a similar prognostic impact for LUAD might serve as a potential panel of prognosis evaluation. However, further validation and modeling of these proteins with other clinical characteristics are needed to judge their comprehensive prognostic impact.

In this study, we employed two statistical strategies (group and paired *t*-test) to screen differentially expressed proteins. Theoretically, statistical strategy one (group *t*-test of two groups) would reveal distinctive features between low-risk and high-risk subtype group, while statistical strategy two (paired *t*-test of tumor and matched normal tissues) would reveal the common and distinctive features of these two groups. We noticed that the differentially expressed proteins between tumor tissues of these two groups did not totally overlap with the distinctively differentially expressed proteins identified from the comparison between tumor and matched normal tissues within them (Fig. 3*B*). We inferred it could be due to the different statistical methods we used and the possible interpretations were as follows: the paired *t*-test considered the relationship between tumor and matched normal tissues in every patient, thus it was more sensitive to minor changes and more resistant to individual differences. But the paired *t*-test considered both *p*-value and significant pairs (which was set to >80%), making the screening criteria stricter than the grouped *t*-test in some circumstances. Therefore, the differentially expressed proteins derived from these two statistical strategies did not overlap completely. To avoid losing potential valuable targets, we included the proteins we identified from these two statistical strategies for interpretation and bioinformatic analysis and considered this would contribute to more comprehensive information.

In the case of PPI network construction (Fig. 4), many proteins in cluster 1 and 2 were related to ECM, such as ECM organization and ECM–receptor interaction. This implied that ECM changed dramatically during the transition procedure from low-risk subtype to high-risk subtype. ECM is composed of a complex mixture of structural and functional biological molecules and is responsible for cell–cell communication, cell adhesion, and proliferation (26). Many important components of ECM, which were differentially expressed in cluster 1 or 2, such as COL6A1, COL1A1, and COL3A1, are mainly secreted by cancer-associated fibroblasts (CAF). CAF are a dominant cell type in tumor stroma and generate a protumorigenic microenvironment (27). CAF and ECM have been extensively studied in recent years (28, 29). In the 2015 World Health Organization Classification of Lung Cancer, the presence of CAF is an indicator of invasion in early-stage LUAD (2). Stromal markers of activated CAF have been proved to predict poor prognosis in non-small-cell lung cancer including adenocarcinoma (30). A previous study indicated that CAF could promote the invasion of LUAD cells by remodeling extracellular matrix (31). Another study also demonstrated that metaplastic breast carcinoma, which is the most lethal subtype of triple-negative breast cancer (TNBC), showed an active ECM compared with other subtypes by quantitative proteomics (32). This hinted that the remodeling of ECM may play a critical role in transition from low-risk subtype to high-risk subtype in early-stage LUAD. Interestingly, MCM complex was one of the significant enriched GO terms in cluster 2. It is formed by minichromosome maintenance (MCM) proteins 2 to 7 and MCM 2 to 5 were the major upregulated MCM family members in cluster 2. MCM complex plays a crucial role in the initiation of DNA synthesis and DNA replication (33). MCM4 has been reported to promote proliferation of LUAD cells and is correlated with Ki-67 and cyclin E expression (34). In addition, MCM7 was reported to have significantly high expression in solid predominant LUAD, which was associated with poor prognosis (35). Additionally, proteins in cluster 2 were significantly enriched in DNA replication and cell cycle (Fig. 4*B*). This indicated that cell proliferation was more active in the high-risk subtype (micropapillary and solid) than in the low-risk subtype (lepidic).

Similarly, in the PPI shown in Figure 5, the distinctive differentially expressed proteins derived from comparison between tumor and matched normal tissues in these two groups were also significantly enriched in ECM organization, ECM proteoglycans, and unwinding of DNA (Fig. 5*B*). Moreover, several enriched terms in GO and KEGG were associated with immune system, such as interferon signaling, complement cascade, and neutrophil degranulation. This indicated that tumor immune microenvironment dysfunction was also pivotal in the transition between different subtypes. Previous studies have verified that lepidic pattern was associated with a less malignant tumor microenvironment (36). Contrarily, solid pattern displays an active cytotoxic immune signature (18). Several other pathways might be also associated with the transition of different subtypes, such as metabolic pathways and vesicle-mediated transport and so on (supplemental Table S5). These indicated that transition procedure between LUAD subtypes is complex and involves numerous biological processes and signaling pathways.

LUADs with high-risk subtypes, namely micropapillary and solid patterns, are reported to be associated with poor prognosis. However, the role of the proteins in cancer invasiveness and their contribution to the prognosis of LUAD remain largely unknown. In the present study, the matched mRNA expression of 63 proteins in cluster 1 to 3 was significantly correlated with prognosis, especially some proteins correlated to ECM remodeling and cell proliferation. High expression of matched mRNAs of the proteins in cluster 1 and 3 was found to predict favorable prognosis, and high expression of matched mRNAs of the proteins in cluster 2 was correlated to poor prognosis. The results were further verified by the IHC analyses of ERO1A and FEN1 in an independent early-stage LUAD cohort, although the result of ERO1A only reached marginal statistical significance (*p* = 0.05). Similarly, previous studies have also demonstrated individual proteins that were aberrantly expressed in specific subtypes that were found to be associated with prognosis, such as MCM7 (35) and AKR1B10 (37). In this study, we found a panel of 63 proteins that might be correlated with prognosis of early-stage LUAD, but it should be verified in a large cohort of lung cancer patients. This panel from proteomics analysis may serve as potential biomarkers for prognostic evaluation in clinical practice. Furthermore, we can construct an efficient prognostic evaluation model based on this panel and clinicopathological characteristics. It may provide more precise information for decision-making.

This study has a few limitations. First, we utilized the RNA-seq data set from TCGA to evaluate the impact of these proteins on prognosis, because there was no database of proteins that provided survival information. Second, we chose only two representative proteins (ERO1A and FEN1) to verify their association with prognosis in the validation experiment using IHC, as the quantity of tissue specimens was limited, and it was not practical to detect the expression of 63 proteins using IHC. Third, there was no complete information about recurrence-free survival (RFS), so we could not evaluate the relationship between these proteins and RFS.

## Summary

In this study, we performed high-quality proteomic analysis of tumor and normal tissues from LUADs with low-risk and high-risk subtypes. We found several differentially expressed proteins and pathways between the two groups. These proteins could be used as diagnosis and prognosis biomarkers. Nevertheless, our study was a preliminary exploration, and further experiments and validations are required to identify the role of differential gene expression in various subtypes of LUAD in its prognosis.

## Institutional Review Board Statement

This study was approved by the Ethics Committee of Peking University Cancer Hospital & Institute (Institutional Review Board No. 2019KT59).

## Data Availability

Mass spectrometry proteomics data have been deposited at the ProteomeXchange Consortium *via* the MassIVE partner repository with the data set identifier PXD020423.

## Conflict of interest

The authors declare no conflict of interest.

## References

[1] Siegel R.L., Miller K.D., Jemal A. (2018). Cancer statistics, 2018. CA Cancer J. Clin..

[2] Travis W.D., Burke A.P. (2015). WHO Classification of Tumours of the Lung, Pleura, Thymus and Heart.

[3] Mäkinen J.M., Laitakari K., Johnson S., Mäkitaro R., Bloigu R., Lappi-Blanco E., Kaarteenaho R. (2015). Nonpredominant lepidic pattern correlates with better outcome in invasive lung adenocarcinoma. Lung Cancer.

[4] Yuan Y., Ma G., Zhang Y., Chen H. (2018). Presence of micropapillary and solid patterns are associated with nodal upstaging and unfavorable prognosis among patient with cT1N0M0 lung adenocarcinoma: A large-scale analysis. J. Cancer Res. Clin. Oncol..

[5] Yoshiya T., Mimae T., Tsutani Y., Tsubokawa N., Sasada S., Miyata Y., Kushitani K., Takeshima Y., Murakami S., Ito H., Nakayama H., Okada M. (2016). Prognostic role of subtype classification in small-sized pathologic N0 invasive lung adenocarcinoma. Ann. Thorac. Surg..

[6] Hung J.J., Jeng W.J., Hsu W.H., Chou T.Y., Huang B.S., Wu Y.C. (2012). Predictors of death, local recurrence, and distant metastasis in completely resected pathological stage-I non-small-cell lung cancer. J. Thorac. Oncol..

[7] Ge S., Xia X., Ding C., Zhen B., Zhou Q., Feng J., Yuan J., Chen R., Li Y., Ge Z., Ji J., Zhang L., Wang J., Li Z., Lai Y. (2018). A proteomic landscape of diffuse-type gastric cancer. Nat. Commun..

[8] Law H.C., Lagundžin D., Clement E.J., Qiao F., Wagner Z.S., Krieger K.L., Costanzo-Garvey D., Caffrey T.C., Grem J.L., DiMaio D.J., Grandgenett P.M., Cook L.M., Fisher K.W., Yu F., Hollingsworth M.A. (2020). The proteomic landscape of pancreatic ductal adenocarcinoma liver metastases identifies molecular subtypes and associations with clinical response. Clin. Cancer Res..

[9] Jiang Y., Sun A., Zhao Y., Ying W., Sun H., Yang X., Xing B., Sun W., Ren L., Hu B., Li C., Zhang L., Qin G., Zhang M., Chen N. (2019). Proteomics identifies new therapeutic targets of early-stage hepatocellular carcinoma. Nature.

[10] Kato Y., Nakamura H., Tojo H., Nomura M., Nagao T., Kawamura T., Kodama T., Ohira T., Ikeda N., Fehniger T., Marko-Varga G., Nishimura T., Kato H. (2015). A proteomic profiling of laser-microdissected lung adenocarcinoma cells of early lepidic-types. Clin. Transl. Med..

[11] Cancer Genome Atlas Research Network (2014). Comprehensive molecular profiling of lung adenocarcinoma. Nature.

[12] Xu J.Y., Zhang C., Wang X., Zhai L., Ma Y., Mao Y., Qian K., Sun C., Liu Z., Jiang S., Wang M., Feng L., Zhao L., Liu P., Wang B. (2020). Integrative proteomic characterization of human lung adenocarcinoma. Cell.

[13] Gillette M.A., Satpathy S., Cao S., Dhanasekaran S.M., Vasaikar S.V., Krug K., Petralia F., Li Y., Liang W.W., Reva B., Krek A., Ji J., Song X., Liu W., Hong R. (2020). Proteogenomic characterization reveals therapeutic vulnerabilities in lung adenocarcinoma. Cell.

[14] Chen Y.J., Roumeliotis T.I., Chang Y.H., Chen C.T., Han C.L., Lin M.H., Chen H.W., Chang G.C., Chang Y.L., Wu C.T., Lin M.W., Hsieh M.S., Wang Y.T., Chen Y.R., Jonassen I. (2020). Proteogenomics of non-smoking lung cancer in East Asia delineates molecular signatures of pathogenesis and progression. Cell.

[15] Detterbeck F.C., Boffa D.J., Kim A.W., Tanoue L.T. (2017). The eighth edition lung cancer stage classification. Chest.

[16] Colaprico A., Silva T.C., Olsen C., Garofano L., Cava C., Garolini D., Sabedot T.S., Malta T.M., Pagnotta S.M., Castiglioni I., Ceccarelli M., Bontempi G., Noushmehr H. (2016). TCGAbiolinks: An R/Bioconductor package for integrative analysis of TCGA data. Nucleic Acids Res..

[17] Murakami S., Ito H., Tsubokawa N., Mimae T., Sasada S., Yoshiya T., Miyata Y., Yokose T., Okada M., Nakayama H. (2015). Prognostic value of the new IASLC/ATS/ERS classification of clinical stage IA lung adenocarcinoma. Lung Cancer.

[18] Dong Z.Y., Zhang C., Li Y.F., Su J., Xie Z., Liu S.Y., Yan L.X., Chen Z.H., Yang X.N., Lin J.T., Tu H.Y., Yang J.J., Zhou Q., Sun Y.L., Zhong W.Z. (2018). Genetic and immune profiles of solid predominant lung adenocarcinoma reveal potential immunotherapeutic strategies. J. Thorac. Oncol..

[19] Naso J.R., Wang G., Pender A., Wong S.K., Zhu J., Ho C., Ionescu D.N., Zhou C. (2020). Intratumoral heterogeneity in programmed death-ligand 1 immunoreactivity is associated with variation in non-small cell lung carcinoma histotype. Histopathology.

[20] Miyazawa T., Marushima H., Saji H., Kojima K., Hoshikawa M., Takagi M., Nakamura H. (2019). PD-L1 expression in non-small-cell lung cancer including various adenocarcinoma subtypes. Ann. Thorac. Cardiovasc. Surg..

[21] Murphy S.J., Wigle D.A., Lima J.F., Harris F.R., Johnson S.H., Halling G., Asiedu M.K., Seto C.T., Terra S., Kosari F., Peikert T., Yang P., Aubry M.C., Vasmatzis G. (2014). Genomic rearrangements define lineage relationships between adjacent lepidic and invasive components in lung adenocarcinoma. Cancer Res..

[22] Mazières J., Rouquette I., Lepage B., Milia J., Brouchet L., Guibert N., Beau-Faller M., Validire P., Hofman P., Fouret P. (2013). Specificities of lung adenocarcinoma in women who have never smoked. J. Thorac. Oncol..

[23] Maeda R., Ishii G., Yoshida J., Hishida T., Nishimura M., Nagai K. (2011). Influence of cigarette smoking on histological subtypes of stage I lung adenocarcinoma. J. Thorac. Oncol..

[24] Chen J., Yang H., Teo A.S.M., Amer L.B., Sherbaf F.G., Tan C.Q., Alvarez J.J.S., Lu B., Lim J.Q., Takano A., Nahar R., Lee Y.Y., Phua C.Z.J., Chua K.P., Suteja L. (2020). Genomic landscape of lung adenocarcinoma in East Asians. Nat. Genet..

[25] Nagahashi M., Sato S., Yuza K., Shimada Y., Ichikawa H., Watanabe S., Takada K., Okamoto T., Okuda S., Lyle S., Takabe K., Tsuchida M., Wakai T. (2018). Common driver mutations and smoking history affect tumor mutation burden in lung adenocarcinoma. J. Surg. Res..

[26] Frantz C., Stewart K.M., Weaver V.M. (2010). The extracellular matrix at a glance. J. Cell Sci..

[27] Kalluri R. (2016). The biology and function of fibroblasts in cancer. Nat. Rev. Cancer.

[28] Walker C., Mojares E., Del Río Hernández A. (2018). Role of extracellular matrix in development and cancer progression. Int. J. Mol. Sci..

[29] Cox T.R., Erler J.T. (2014). Molecular pathways: Connecting fibrosis and solid tumor metastasis. Clin. Cancer Res..

[30] Alcaraz J., Carrasco J.L., Millares L., Luis I.C., Fernández-Porras F.J., Martínez-Romero A., Diaz-Valdivia N., De Cos J.S., Rami-Porta R., Seijo L., Ramírez J., Pajares M.J., Reguart N., Barreiro E., Monsó E. (2019). Stromal markers of activated tumor associated fibroblasts predict poor survival and are associated with necrosis in non-small cell lung cancer. Lung Cancer.

[31] Neri S., Hashimoto H., Kii H., Watanabe H., Masutomi K., Kuwata T., Date H., Tsuboi M., Goto K., Ochiai A., Ishii G. (2016). Cancer cell invasion driven by extracellular matrix remodeling is dependent on the properties of cancer-associated fibroblasts. J. Cancer Res. Clin. Oncol..

[32] Djomehri S.I., Gonzalez M.E., da Veiga Leprevost F., Tekula S.R., Chang H.Y., White M.J., Cimino-Mathews A., Burman B., Basrur V., Argani P., Nesvizhskii A.I., Kleer C.G. (2020). Quantitative proteomic landscape of metaplastic breast carcinoma pathological subtypes and their relationship to triple-negative tumors. Nat. Commun..

[33] Ishimi Y. (2018). Regulation of MCM2-7 function. Genes Genet. Syst..

[34] Kikuchi J., Kinoshita I., Shimizu Y., Kikuchi E., Takeda K., Aburatani H., Oizumi S., Konishi J., Kaga K., Matsuno Y., Birrer M.J., Nishimura M., Dosaka-Akita H. (2011). Minichromosome maintenance (MCM) protein 4 as a marker for proliferation and its clinical and clinicopathological significance in non-small cell lung cancer. Lung Cancer.

[35] Wang H., Zhou C., Su B., Teng G., Zheng Y., Zhou X., Guo L., Xu F., Wang X. (2017). MCM7 expression is correlated with histological subtypes of lung adenocarcinoma and predictive of poor prognosis. Int. J. Clin. Exp. Pathol..

[36] Katsumata S., Aokage K., Miyoshi T., Tane K., Nakamura H., Sugano M., Kojima M., Fujii S., Kuwata T., Ochiai A., Hayashi R., Tsuboi M., Ishii G. (2018). Differences of tumor microenvironment between stage I lepidic-positive and lepidic-negative lung adenocarcinomas. J. Thorac. Cardiovasc. Surg..

[37] Hung J.J., Yeh Y.C., Hsu W.H. (2018). Prognostic significance of AKR1B10 in patients with resected lung adenocarcinoma. Thorac. Cancer.

