# Supplementary material for: Proteomic Analyses Identify Differentially Expressed Proteins and Pathways Between Low-Risk and High-Risk Subtypes of Early-Stage Lung Adenocarcinoma and Their Prognostic Impacts
Source: Mol Cell Proteomics. 2021 Jan 26;20:100015. doi: 10.1074/mcp.RA120.002384 (PMC7950210; doi:10.1074/mcp.RA120.002384)
Supplement: Supplemental Figures 1–3 [file mmc1.docx]

**Supplementary materials for**

**Proteomic analyses identify differentially expressed proteins and pathways between low-risk and high-risk subtypes of early-stage lung adenocarcinoma and their prognostic impacts**

Juntuo Zhou^2,*^, Bing Liu^1,*^, Zhongwu Li^3^, Yang Li^4^, Xi Chen^4^, Yuanyuan Ma^1^, Shi Yan^1^, Xin Yang^3^, Lijun Zhong^5,#^ and Nan Wu^1,#^

^1^ Key Laboratory of Carcinogenesis and Translational Research (Ministry of Education), Department of Thoracic Surgery II, Peking University Cancer Hospital & Institute, Beijing 100142, China

^2^ Beijing Advanced Innovation Center for Big Data-Based Precision Medicine, Beihang University, Beijing 100083, China

^3^ Key Laboratory of Carcinogenesis and Translational Research (Ministry of Education), Department of Pathology, Peking University Cancer Hospital & Institute, Beijing 100142, China

^4^ Department of Pathology, Peking University Health Science Center, Beijing 100191, China

^5^ Center of Medical and Health Analysis, Peking University Health Science Center, Beijing 100191, China

**Figure S1. Multi-scatter plots of QC samples of the proteomic analysis.**


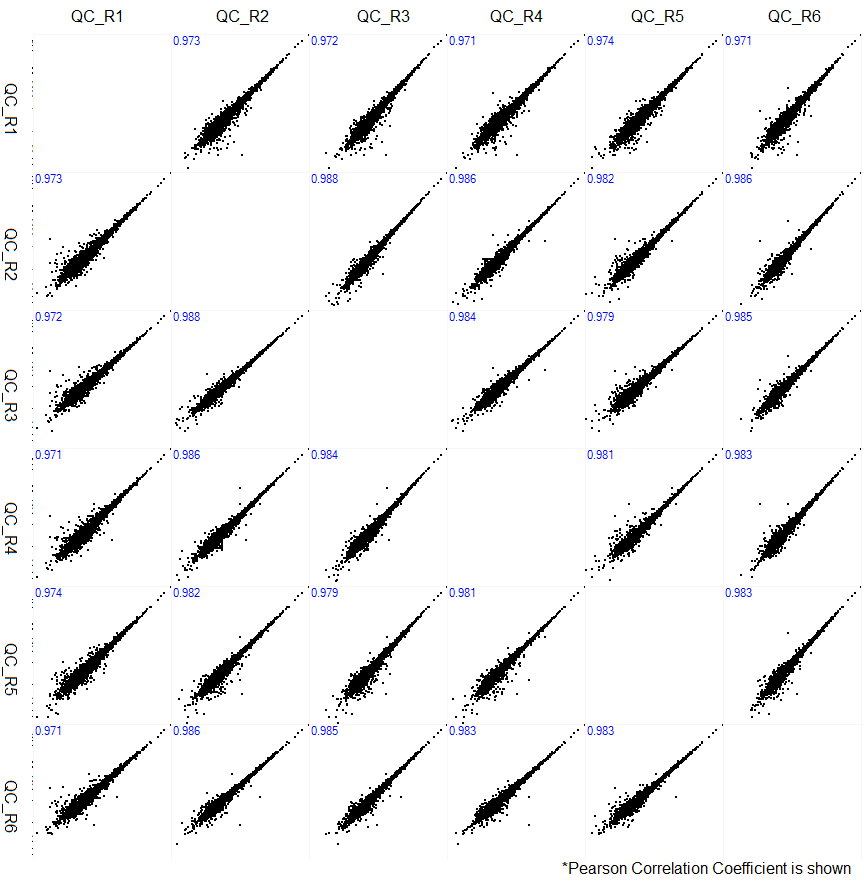


**Figure S1. Multi-scatter plots of QC samples of the proteomic analysis.**

Multi-scatter plots of QC samples of the proteomic analysis are shown. 5926 proteins were included, and log2 transformation was performed for the protein intensity before analysis.

**Figure S2. Hierarchical clustering heat map presenting the relevance between EGFR mutation and LUAD subtypes.**


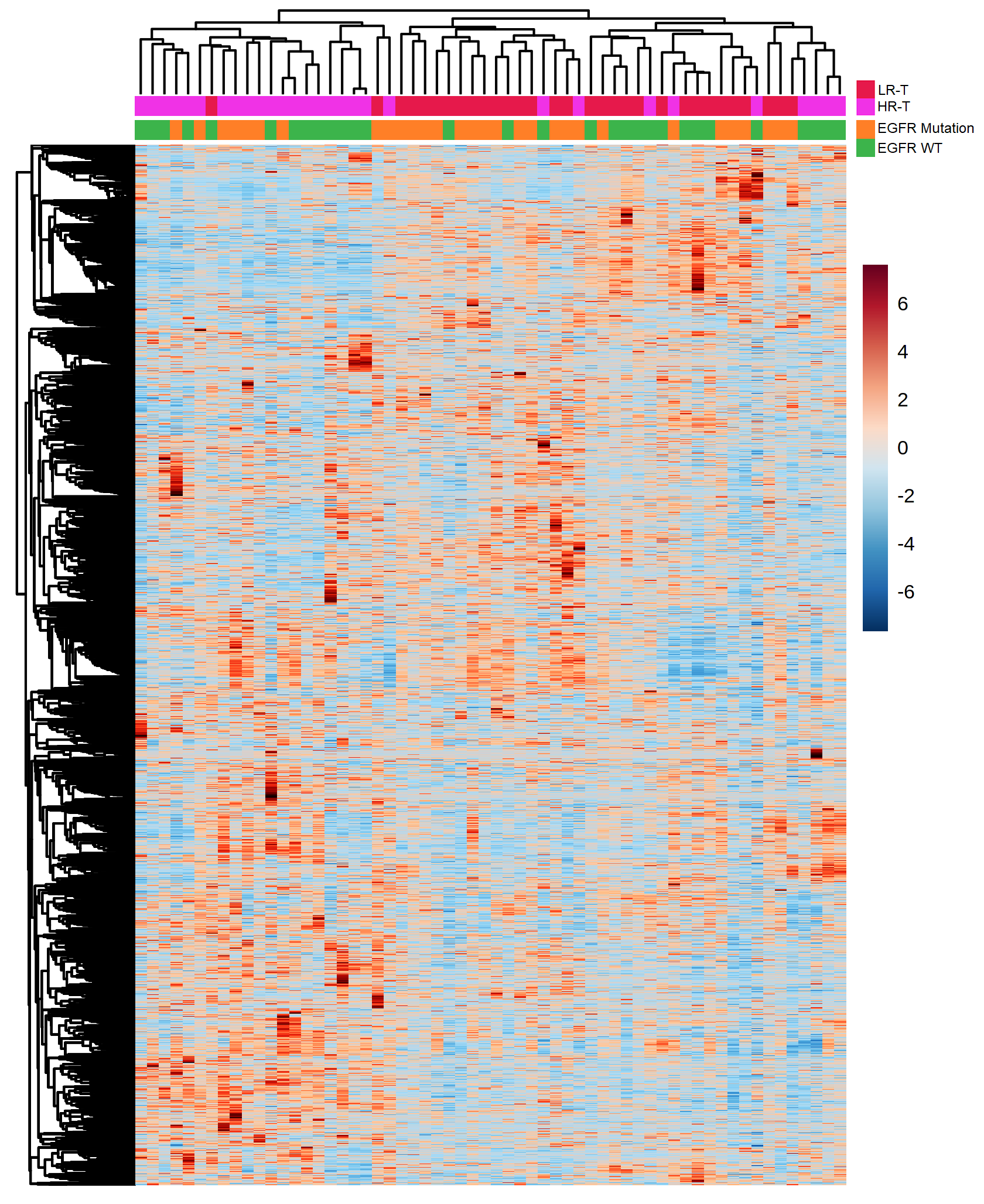


**Figure S2. Hierarchical clustering heat map presenting the relevance between EGFR mutation and LUAD subtypes.**

Hierarchical clustering analysis was performed by for tumor samples by MetaboAnalyst web service. Pearson correlation was used. EGFR mutation is not relevant to the proteomic classification of the subtypes, as presented by the colored label (red, LR-T; pink, HR-T; orange, EGFR mutation; green, EGFR wildtype).

**Figure S3. Hierarchical clustering heat map presenting the relevance between TNM stage and LUAD subtypes.**


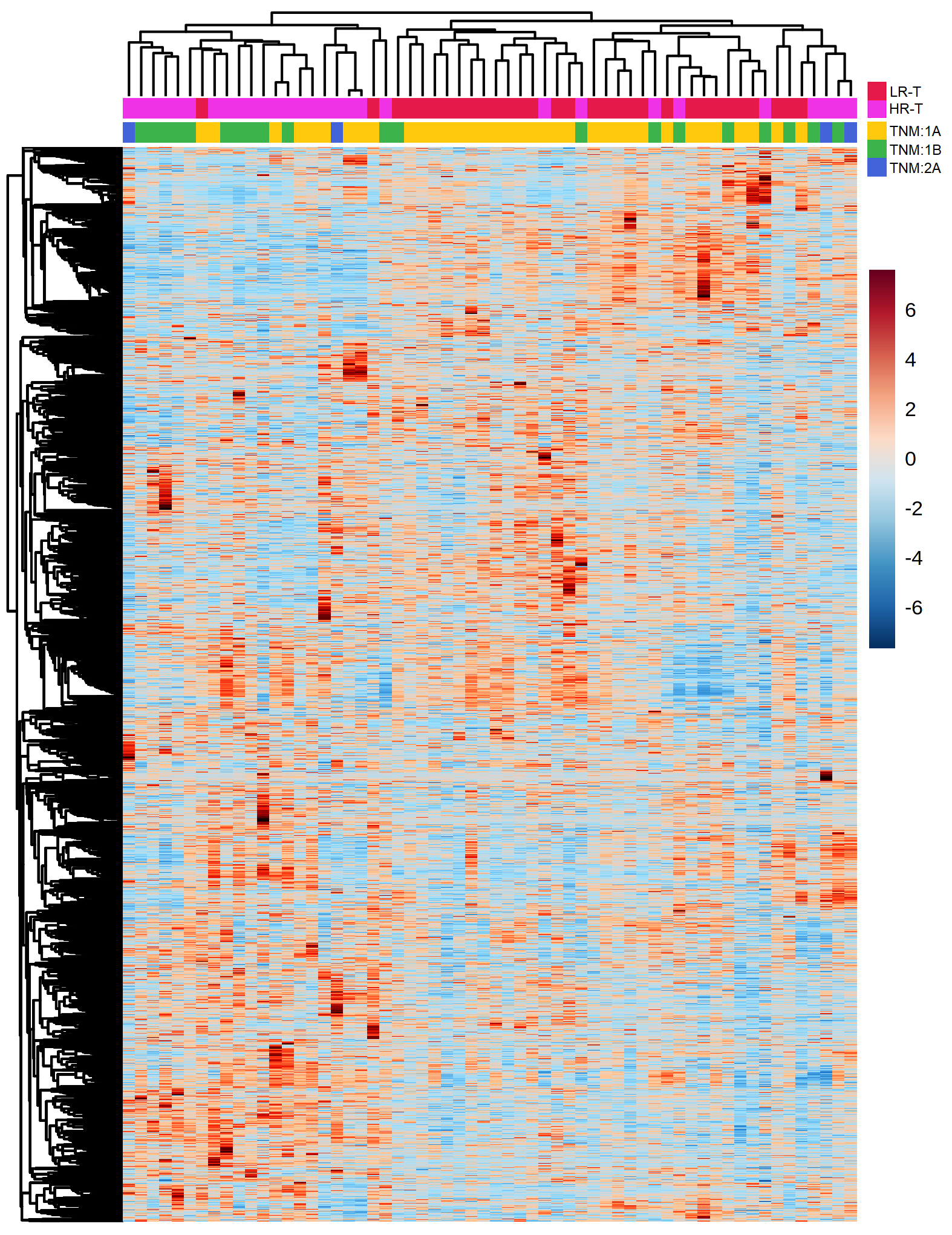


**Figure S3. Hierarchical clustering heat map presenting the relevance between TNM stage and LUAD subtypes.**

Hierarchical clustering analysis was performed by for tumor samples by MetaboAnalyst web service. Pearson correlation was used. TNM stage is not relevant to the proteomic classification of the subtypes, as presented by the colored label (red, LR-T; pink, HR-T; yellow, TNM1A; green, TNM1B; blue, TNM2A).
